# Super-Resolution for Improving EEG Spatial Resolution using Deep Convolutional Neural Network—Feasibility Study

**DOI:** 10.3390/s19235317

**Published:** 2019-12-03

**Authors:** Moonyoung Kwon, Sangjun Han, Kiwoong Kim, Sung Chan Jun

**Affiliations:** 1 School of Electrical Engineering and Computer Science, Gwangju Institute of Science and Technology, Gwangju 61005, Korea; mykwon@gist.ac.kr; 2 AI Core Development Team, LG Electronics, Seoul 07796, Korea; sjun.han@lgsp.co.kr; 3 Center for Biosignals, Korea Research institute of Science and Standards, Daejeon 34113, Korea; kwkim@kriss.re.kr; 4 Department of Medical Physics, University of Science and Technology, Daejeon 34113, Korea

**Keywords:** convolutional neural networks, electroencephalography, spatial resolution, super-resolution

## Abstract

Electroencephalography (EEG) has relatively poor spatial resolution and may yield incorrect brain dynamics and distort topography; thus, high-density EEG systems are necessary for better analysis. Conventional methods have been proposed to solve these problems, however, they depend on parameters or brain models that are not simple to address. Therefore, new approaches are necessary to enhance EEG spatial resolution while maintaining its data properties. In this work, we investigated the super-resolution (SR) technique using deep convolutional neural networks (CNN) with simulated EEG data with white Gaussian and real brain noises, and experimental EEG data obtained during an auditory evoked potential task. SR EEG simulated data with white Gaussian noise or brain noise demonstrated a lower mean squared error and higher correlations with sensor information, and detected sources even more clearly than did low resolution (LR) EEG. In addition, experimental SR data also demonstrated far smaller errors for N1 and P2 components, and yielded reasonable localized sources, while LR data did not. We verified our proposed approach’s feasibility and efficacy, and conclude that it may be possible to explore various brain dynamics even with a small number of sensors.

## 1. Introduction

Super-resolution (SR) is a technique that enhances low-resolution (LR) images’ quality to high-resolution (HR). Recently, this underdetermined inverse problem in imaging was addressed successfully by a data-driven approach, deep convolutional neural networks (CNN). Firstly, Dong et al., (2015) suggested simple neural networks using three convolution operations [1], and Kim et al., (2015) extended the model by including twenty layers with larger kernels [2] that learned image residuals to increase convergence speed. In subsequent work, Kim and his colleagues constructed a recurrent model of convolution layers and adopted the skip-connection to prevent the vanishing gradient problem [3]. To convert LR to HR efficiently, Shi et al., (2016) devised sub-pixel convolutions rather than handcrafted bi-cubic interpolation in the initial layer [4]. Further, a novel approach that minimizes loss related to a pre-trained model feature rather than the mean squared error was proposed to recover high-frequency details and match humans’ visual perception [5]. It has been assumed that SR images may be estimated in real image distributions composed of generative adversarial networks (GAN). In [6], features from several local-residual-dense blocks were concatenated with point-wise convolution, and up-scaled using sub-pixel convolution. We found previously that residual and dense structures guarantee good classification performance by propagating gradients well [7,8]. In audio processing, SR may be understood as a concept of generative signal modeling. Audio SR using CNN may increase the signal’s sampling rate and predict missing samples in LR signals [9]. With respect to the signal-to-noise ratio (SNR) and log spectral distance, the CNN-based audio SR outperformed spline interpolation and artificial neural networks (ANN).

Electroencephalography (EEG) measures electrical potentials from sensors attached to the scalp. Cooperating neurons in the brain’s cortices generate brain waves, and these electrical activities are widespread throughout the cerebrospinal fluid (CSF), skull, and scalp. Compared to other brain imaging techniques, EEG has a relatively good temporal resolution, but poor spatial resolution that generates inaccurate source information on the cortex [10,11], distorts topographical maps by removing high spatial frequency [12,13], and makes it difficult to reject artefacts as discriminable independent components [14]. Therefore, although they are more costly, high-density EEG systems are required to reduce the errors in estimating brain function.

To address the disadvantage of poor spatial resolution, many researchers have studied methods that enhance EEG spatial resolution to reduce signal distortion. Hjorth (1975) introduced a local surface Laplacian method that estimated scalp potentials from four neighboring sensors’ averaged potentials [15], and its modified surface Laplacian [16]. However, local surface Laplacian methods are vulnerable to eye movements and blinking [17]. Thus, Perrin et al., (1987) and Nunez (1995) proposed a global surface Laplacian by computing second derivatives of interpolated values [18,19]. In addition, spherical harmonics that solve the partial differential equations on an orthogonal basis [20] were reported. However, a spherical spline is sensitive to spline parameters [21]. Another approach is a cortical imaging method or current source density that estimates scalp voltages from current dipoles using a volume conduction model [22,23,24,25]; however, these are severely model-dependent, and demonstrate uncertainty with unknown sources [26]. Accordingly, there has been almost no breakthrough to date that improves EEG spatial information, although modified methods have been explored [27]. Recently, compressed sensing that recovers original data from far fewer features or measurements seems promising [28,29]. Compressive sensing was applied to wireless EEG systems for home care that may monitor the progression of Alzheimer’s disease and the effect of drugs [30,31], but its application depends on the original data’s redundancy [28,29]. However, the recent success in applying deep learning to the SR technique may offer a way to overcome EEG’s low resolution inexpensively.

Corley et al., (2018) investigated the EEG SR technique first in mental imagery classification in brain–computer interface (BCI) [32]. They selected 8 or 16 channels from the original 32 and then up-scaled them spatially to the original 32 channels using Wasserstein GAN; SR performance was evaluated to determine whether SR data may be classified to the same degree as the original data, and SR was found to yield comparable EEG classification results. However, it was not possible to determine whether SR recovers or maintains the original EEG signals’ characteristics.

In this work, we proposed deep CNN firstly to enhance EEG data’s spatial resolution and investigated its feasibility in sensor and source aspects. Recovered high-density EEG data could be applied not only to detect events at the sensor level, but also to detect the source; further, it may estimate functional connectivity and capture a subject’s intention and mental states. We investigated 2-, 4-, 8-, and 16-fold scale-ups over various SNRs extensively. We note that this work was an extended version of our IEEE SMC 2018 conference paper [33] that reported the preliminary results of SR CNN models for simulated data. However, this work reports a more extensive and in-depth investigation with both simulated and experimental data. In addition, the up-scaling direction and evaluations were considered using deep learning and signal processing approaches. To observe SR EEG characteristics quantitatively, the mean squared error (MSE) and correlation at the sensor level, as well as source localization at the source level, were considered. Section 2 presents details of the simulated and experimental data and introduces our proposed CNN structure. In Section 3, we present the evaluation of the SR data characteristics for the simulated and experimental data. SR-related issues and limitations of this work are discussed in Section 4, and Section 5 provides our conclusions.

## 2. Materials and Methods

### 2.1. Simulated Data

**Figure 1:**
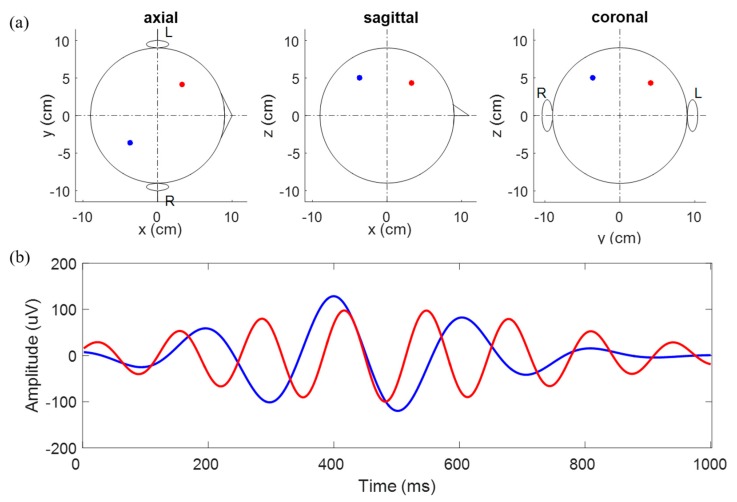
Simulated data without noise (**a**) position, (**b**) time series of two dipoles.

To generate simulated EEG data, we considered a three-spherical shell head model that represents the brain (innermost), and skull and scalp (outermost) with their respective conductivities, 1, 0.0125, and 1 [34]. Each shell’s relative radii were 0.87, 0.92, and 1, respectively. The boundary element method (BEM) was applied to compute the simulated EEG data. All EEG sensors were placed on the outermost shell (representing the scalp) according to the 10–10 international system. Two dipoles within the brain were considered and BEM was applied to compute the EEG data at the sensors. Simulated data were sampled at 512 Hz, and each trial lasted 1 s. To investigate the SR approach’s feasibility using CNN, dipoles in the simulation were chosen to be more realistic, but to determine source localization simply. A single dipole is very simple, while more than three make it difficult to estimate the source’s performance. Thus, we considered two dipoles in this study, in which each is located in each hemisphere sufficiently far apart to localize reasonably at low SNRs. In reality, two dipoles are known to explain experimental auditory evoked potential (AEP) data. Noiseless simulated data are illustrated in Figure 1. Finally, to generate noisy data, we added white Gaussian noise or real brain noise (eyes open resting state) to the EEG computed, and generated various SNR data by scaling the noise data (SNR of 100, 50, 10, 5, 1, 0.5, 0.1, 0.05, and 0.01). We note that real brain noise was acquired from one subject during a two-minute resting state (eyes open) without eye movements using the Biosemi Active Two system with 64 channels.

### 2.2. Experimental Data

We collected EEG data (512 Hz) from six subjects (aged 26.27 ± 1.03) using the 64 channel Biosemi ActiveTwo system (Amsterdam, Netherlands) during an experiment that the Institutional Review Board of Gwangju Institute of Science and Technology approved (20181023-HR-39-02-02). Further, two re-referencing earlobe channels were used, and electrooculogram (EOG) and electromyogram (EMG) data were collected to monitor unexpected noise from the eyes, jaw, and chin. Subjects fixed their gaze on the monitor for one minute and closed their eyes for one minute. Thereafter, they performed the AEP task for five runs, each of which consisted of 200 trials. Finally, we collected eyes-open and eyes-closed data again. During the AEP task, an auditory stimulus, a beeping sound 50 ms in duration with a 1000 Hz square wave tone, was given for each trial, and subjects were instructed to concentrate on the sound. An inter-stimulus interval (ISI) between 1000 and 1500 ms was given randomly. The EEG data from −300 to 1000 ms were used and preprocessed by band-pass filtering from 1 to 50 Hz. Severe noise components were discarded using independent component analysis (ICA), and bad trials were rejected by visual inspection. We observed that a small number of trials were contaminated by severe noise attributable to frowning or unexpected spike signals. The data were divided randomly into training (64%), validation (16%), and test (20%) trials, and the CNN process and evaluation were conducted with these sets. This procedure was repeated five times with different training and test sets for cross-validation.

After checking the six subjects’ data quality, one subject’s data were discarded because of unexpected severe noise. The CNN method for super-resolution was applied to the five datasets remaining, and we found that all data yielded similar trends. Because our goal in this work was to investigate the feasibility of the SR approach using CNN, we believed that one subject’s data were sufficiently good for deep analysis, and thus, we selected one of the five datasets that demonstrated less noisy trials in this work, although no significant difference in the data’s quality was seen. We rejected noisy components and trials from the selected subject’s data and finally used 932 among 1000 trials in our analysis.

### 2.3. Generating Low Spatial Resolution Data

First, we defined HR, LR, and SR. HR represented the original data (64 channels) and was used for comparison with the CNN output in the training step. LR represented 64-channel data interpolated from 32-, 16-, 8-, and 4-channel data, and was used for CNN input data. Throughout this work, we often addressed LR without interpolation, which represents simply the original EEG data with 32, 16, 8, or 4 channels to compare the source localization results. SR is the output of the CNN process, i.e., the super-resolution data (64 channels).

**Figure 2:**
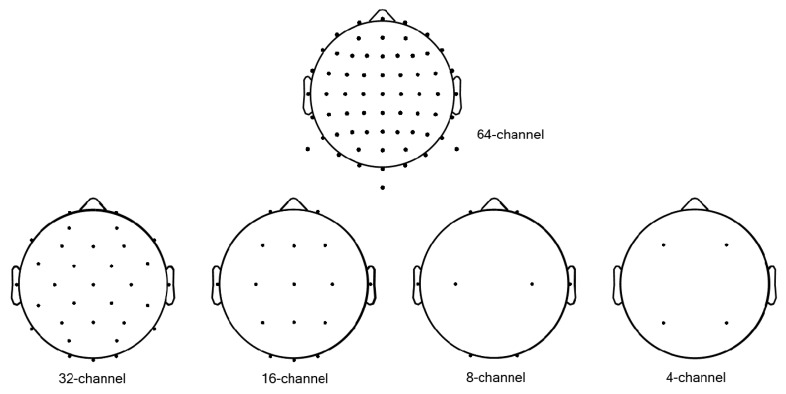
Channel locations.

**Figure 3:**
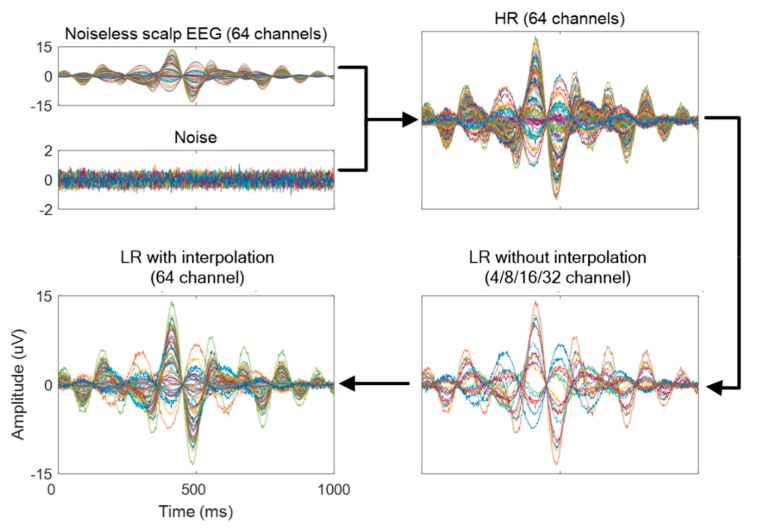
Generation of low resolution (LR) data with white Gaussian noise (signal-to-noise ratio (SNR) of 100) and processing high resolution (HR) and LR data when up-scaling from 16→64 (4×).

In the SR study, we down-scaled the original EEG data (64 channels) 2, 4, 8, and 16 times; i.e., 32 (64→32), 16 (64→16), 8 (64→8), and 4 (64→4) channel data were generated. Our channel configuration was based on the Biosemi ActiveTwo 64-channel system’s configuration (https://www.biosemi.com/headcap.htm). Then, we reduced these 64 channels to 32, 16, 8, and 4. We chose the 32 and 16 channels based on the Biosemi configuration system; 8 and 4 channels were chosen among 64 channels, and remained positioned evenly on the head. Details of the channels chosen are shown in Figure 2. Then, we investigated two SR approaches (scale-up to 64-channel data) from the downscaled data as follows:

- Conventional interpolation approach (LR): Data for missing channels were estimated from known nearby channels by simple linear interpolation (Figure 3). We note that this simple transformation of data with fewer channels (4-, 8-, 16-, or 32-channel data) to interpolated LR data (64-channel data) is a fundamentally ill-posed problem; Dong et al., up-scaled their input LR images to the size desired using bicubic interpolation to achieve good beginning initialization [1].

Our proposed deep CNN approach (SR): Input data are introduced as the data (interpolated LR) estimated by the conventional interpolation approach above. Then, trained deep CNN estimates the corresponding SR data from the input data given. Detailed information on CNN is described in the following section.

### 2.4. Deep CNN for Super-Resolution

**Figure 4:**
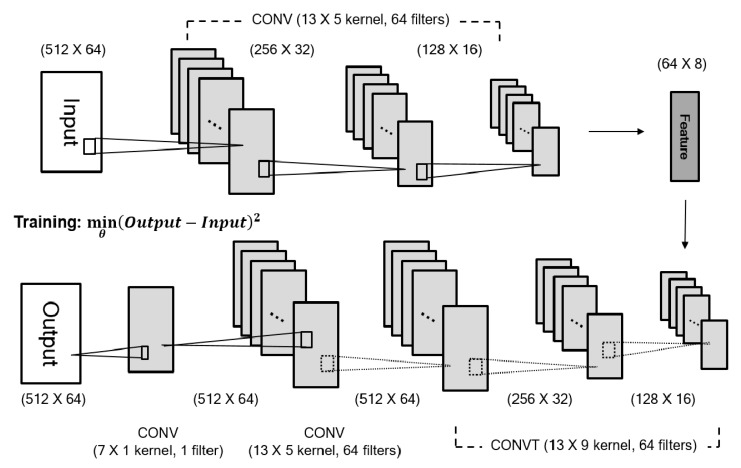
Proposed deep convolutional neural network (CNN) for super-resolution. Input and output size are represented for simulated data. CONV: convolution, CONVT: transposed convolution.

The deep CNN structure that we designed adopted the symmetry of a stacked denoising autoencoder [35] (Figure 4) and consists of three components: encoder, decoder, and integration. The encoder, devised for down-sampling the input LR data into latent space, conducts three successive convolutions (13 × 5 kernel, 64 filters, 2 strides). From the latent space, the decoder up-samples its data size by applying three transposed convolutions (13 × 9 kernel, 64 filters, 2 up-sampling strides). It is known that transposed convolution may be regarded as a reverse operation of convolution with learnable parameters [36]. However, we verified checkerboard artifacts in the up-sampled data as mentioned in [37]. The integration step is used to prevent any patterned noise and made the data size fit our output HR. Two convolution layers (13 × 5 kernel with 64 filters, 1 stride, and 7 × 1 kernel, 1 filter, 1 stride) were used. Our kernel configurations were chosen because of Kim et al.,’s previous work, which reported that the larger kernel yields higher performance in image SR (13 × 13 vs. 41 × 41) [2]. Further, many studies have tended to separate neural networks into spatial (1 × *m* kernel) and temporal (*n* × 1 kernel) elements [38,39,40], and temporal size was far larger than channel size (up to 8 to 10 times approximately) in our data. Therefore, we chose *n* × *m* kernels (*n* > *m*) to extract spatiotemporal features and the other parameters empirically, including the number of filters and the stride sizes. We observed that using non-linear functions, such as ReLU and tanh led to failed optimization because of its limited function’s value ranges (discussed later); therefore, the linear activation function (*y = x*) was used in this study [41]. The initialization values that He et al. [42] introduced were applied to all layers, and the Adam optimizer [43] was used with an empirical learning rate of 5 × 10^−4^. As stopping criteria for training, we set maximum iteration numbers in which the difference in loss between previous and current steps is so small that it could not lead to overfitting. The maximum iteration numbers were 40 (SNR of 100, 50), 80 (SNR or 10, 5), 150 (SNR of 1), 200 (SNR of 0.5, 0.1), 500 (SNR of 0.05, 0.01) in simulated data and 300 in experimental data. In all cases (noise type and its SNR), the same CNN structure was applied except for the input dimensions (simulated data: 512 × 64, experimental data: 666 × 64). In simulated data, 640 trials were used for training, 160 for validation, and 200 for testing. Further, 596 trials for training, 150 trials for validation, and 186 trials for testing were applied to CNN in the experimental data (932 trials).

### 2.5. Evaluation

The SR data recovered were evaluated with several metrics that differed depending upon the tasks given.

#### 2.5.1. Simulated Data

We compared the LR, HR, and SR data to the original noiseless EEG data for each trial. In simulated data, we know the ground truth, which is noiseless EEG data. Thus, conventionally, a comparison with ground truth was performed. For comparison, the MSE and correlations were computed between the estimated data and the noiseless EEG data at the sensor level for all test trials. At the source level, amplitude error, localization error, and focality were estimated. In source localization, the three-spherical shell head model and BEM were used for forward computing and the array-gain-minimum-variance beamformer was used to perform source localization [44]. The brain region was beamforming-scanned at a 5 mm scanning interval within 51,127 voxels.

- Sensor Level Metrics
  ▪ Mean Squared Error (MSE):
    (1) $$MSE=\frac{1}{t}(\overset{t}{\underset{i=1}{\sum }}{\left(Noiseless_{i}-D_{i}\right)}^{2}$$
    t:the number of time sample, D:HR, LR, or SR
  ▪ Pearson Correlation Coefficient:
    (2) $$\rho_{Noiseless,D}=\;\frac{COV\left(Noiseless,\;D\right)}{\sigma_{Noiseless}\times \sigma_{D}}$$
    COV:covariance, σ:standard deviation
- Source Level (localization) Metrics
  The evaluation metrics were calculated using correct source detection trials at the source level.
  ▪ Amplitude Error:
    (3) $$Amplitude\;Error=\;|Max(Amp_{Noiseles)}-Max(Amp_{D})|$$
    Max(Amp):maximum amplitude in region of interest (ROI), D:HR, LR, or SR
  ▪ Localization Error:
    (4) $$Localization\;Error={\left(pos{\left(x,y,z\right)}_{Noiseless}-pos{\left(x^{\prime },y^{\prime },z^{\prime }\right)}_{D}\right)}^{1/2}$$
    pos(x,y,z):position at maximum amplitude voxel, D:HR, LR, or SR
  ▪ Focality of Localization:
    (5) $$Focality=\frac{1}{the\;number\;of\;voxels\;activated\;(power>threshold)}$$

Localized sources’ activation (magnitude of source) was normalized for comparison. We observed very few small values (weak activation; activation < 0.3) that were quite noisy. For visibility, we set the threshold values to 0.3.

#### 2.5.2. Experimental Data

Event-related potential (ERP) components were calculated at the sensor level. Unlike the simulated data, the experimental data were analyzed using trial averaging data because AEP data are analyzed in this way generally and clear ERP components can locate them with averaged data over trials. Specifically, the N1 and P2 components are known widely to be AEP components’ representative patterns [45,46]. Therefore, the N1 and P2 components’ amplitudes and latencies were estimated in each of the HR, LR, and SR datasets. Similar to the simulated data, localization performance was compared among them according to the number of sources detected and localization amplitude error. Focality was excluded from the experimental dataset because it activated only one voxel per source. In the experimental data, we assumed that the HR data constituted the ground truth because experimental data could not obtain noiseless data.

- Sensor Level Metrics
  ▪ Amplitude Error:
    (6) $$Amplitude\;Error=\;\sum_{k=N1,P2}|Max(Amp_{HR_{k}})-Max(Amp_{D_{k}})|$$
    Max(Amp):maximum amplitude in ROI, D:LR, or SR
  ▪ Latency Error:
    (7) $$Latency\;Error=\;\sum_{k=N1,P2}\left|L_{HR_{k}}-L_{D_{k}}\right|$$
    L:latency at maximum amplitude voxel, D:LR, or SR
  Statistical test: We conducted a statistical analysis (at time point) between LR and HR, and SR and HR for the experimental data. Test data were down-sampled temporally by 8 for simplicity, after which a paired Student’s *t*-test was performed for each time sample. Statistical results at AFz, CPz, TP7, TP8, and POz channels were compared. The statistically different time points (uncorrected, *p* < 0.01) indicated that LR and SR data failed to follow the HR data.
- Source Level (localization) Metrics
  Detected sources were quite focal in several regions and were activated strongly or weakly depending on conditions, while small activation values were observed in other regions (largely, activation < 0.1). From this observation, we set 0.1 as a power threshold empirically because of its visibility in the AEP data.
  ▪ Amplitude Error:
    (8) $$Amplitude\;Error=\;|Max{\left(Amp\right)}_{HR}-Max(Amp_{D})|$$
    Max(Amp):maximum amplitude in ROI, D:LR, or SR
  ▪ The number of error sources: When HR data detect the source at a specific voxel, but LR or SR data did not detect sources at the voxels, then the sources count as an error source. The opposite case also includes error sources.

## 3. Results

### 3.1. SR Results for Simulated Data (White Gaussian Noise)

**Figure 5:**
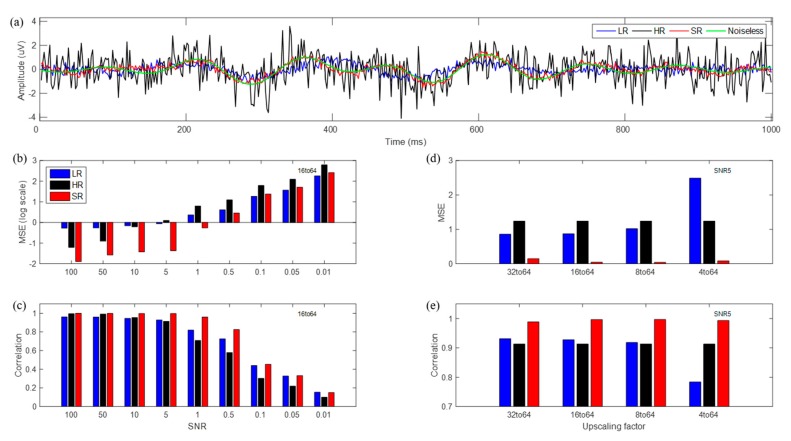
(**a**) Time series of LR (blue), HR (black), super resolution (SR) (red), and noiseless (green) signal for single trial with white Gaussian noise with an SNR of 5 at CPz channel; (**b**) logarithm of mean squared error (MSE) from noiseless signals; (**c**) correlation values according to the SNR with 16→64 up-scaling factor; (**d**) MSE; (**e**) correlation values according to up-scaling factors with an SNR of 5.

Data for one trial at the CPz channel are shown in Figure 5a; the LR data did not follow the original data during some time periods, while the SR data maintained the original data’s trends and generated far less noisy data, unlike the original signal.

Figure 5 presents MSE and correlation coefficients between noiseless EEG data and LR, HR, or SR estimated data at the 16→64 scale-up over varying SNRs. Further, four different scale-up cases, 32→64 (2×), 16→64 (4×), 8→64 (8×), and 4→64 (16×), were compared at an SNR of 5. MSE in HR represented only the noise magnitude over SNRs for comparison with noiseless data. SR demonstrated far smaller MSEs for high SNRs (≥0.5) than did the others, while LR demonstrated smaller MSEs for low SNRs (≤0.1) than did HR and SR. For various scale-ups, SR showed the best performance by far in MSE at high SNRs; LR showed nearly uniform MSEs, except for the 4→64 scale-up. In MSE estimation, sensors on the boundary of head coverage were the principal factors; these sensors’ estimation yielded higher MSEs than did others. We observed that interpolated data at boundary sensors yielded relatively smaller errors with the CNN process up to eight channels, which could cover the boundary of the head as a whole. However, four channels were positioned on central regions and could not cover the boundary; thus, estimated data at boundary sensors could not be optimized sufficiently. Therefore, the 4→64 scale-up yielded relatively larger MSE than did other scale-ups.

With respect to the correlations, SR demonstrated a more notable difference (higher correlation) over 0.5–10 SNRs than did the others; SR had the highest correlations by far for various scale-ups, while LR had nearly uniform correlations except for the 4→64 scale-up. Overall, SR outperformed LR, and even HR, both in MSE and correlations. We note that SR data reduced noise, and thereby, demonstrated higher performance than did the original HR data. Further, SR data followed the trend of noiseless EEG data more closely, although they showed slightly fluctuating high-frequency behavior attributable to the noise’s learning effect. In particular, SR that yielded 20 times less error in MSE than LR was estimated roughly from actual MSE values (LR: 0.87, SR: 0.04), and SR with a 6% higher correlation than LR was estimated from actual correlation values (LR: 0.93, SR: 0.99) at an SNR of 5 and the 16→64 up-scale.

**Figure 6:**
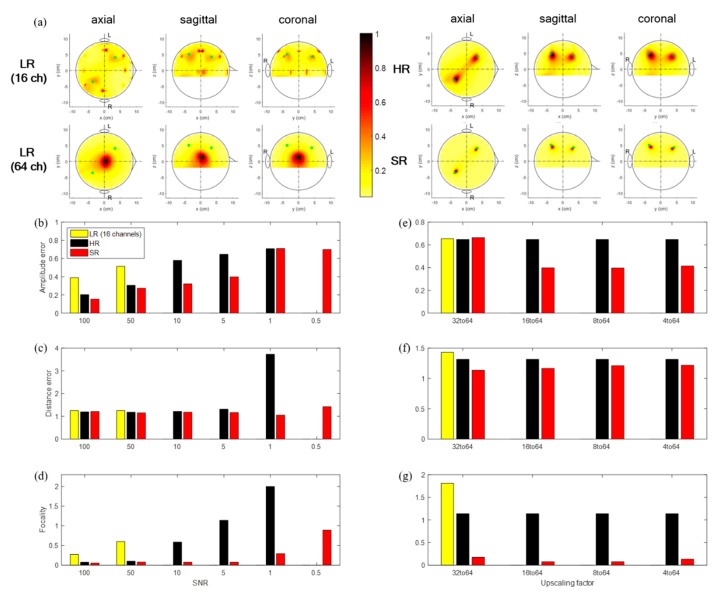
(**a**) Topographic of source localization using LR (16 channels; no interpolation), interpolated LR, HR, SR signal for single trial with white Gaussian noise with an SNR of 5, exact source location (green dot); (**b**,**c**,**d**) amplitude error, distance error, and focality according to SNR with 16→64 up-scaling factor; (**e**,**f**,**g**) amplitude error, distance error, and focality according to up-scaling factor with SNR of 5.

We conducted a source-level analysis for all cases of LR, HR, and SR data, as shown in Figure 6. The source localization results were compared with respect to source detection, amplitudes of sources detected, localization error (distance between maximum voxel and exact source), and source focality. For comparison purposes, source localization was applied to the LR data without interpolation (smaller number of channels than the original 64 channels). We observed that for very low SNRs (≤0.1), none of the LR, HR, and SR data were localized well, in that notable sources were not detected. We compared localization performance over various SNRs and scale-ups for reasonably high SNRs (≥0.5) as shown in Figure 6b–g.

Among the three, the SR data showed the best localization performance in all respects (source detection, source amplitude error, localization error, and focality). LR without interpolated data was localized only for SNRs of 100 or 50, while interpolated LR data did not detect any sources for all SNRs; it is interesting that for an SNR of 0.5, HR data were not localized; however, SR data were localized reasonably well. Two sources were localized in the HR data for all trials over all high SNRs (≥5), while for several trials, sources were not detected at an SNR of 1. Two sources were localized in SR data for all trials over all SNRs (≥0.5). The sources’ amplitude error and focality increased as the SNR decreased, and they demonstrated nearly uniform results over various scale-ups. Localization errors demonstrated marginal values over SNRs and scale-ups. The SR approach showed the best performance by far at both the sensor and source levels, and in particular, SR demonstrated a higher SNR than did the original data. For the 16→64 up-scale at an SNR of 5, SR had 40%, and 12% fewer errors in amplitude and localization, respectively, and SR was 19 times more focal to sources than was LR without interpolation (LR without interpolation: 0.65, 1.32, 1.14, SR: 0.40, 1.16, 0.06).

### 3.2. SR Results for Simulated Data (Real Brain Noise)

**Figure 7:**
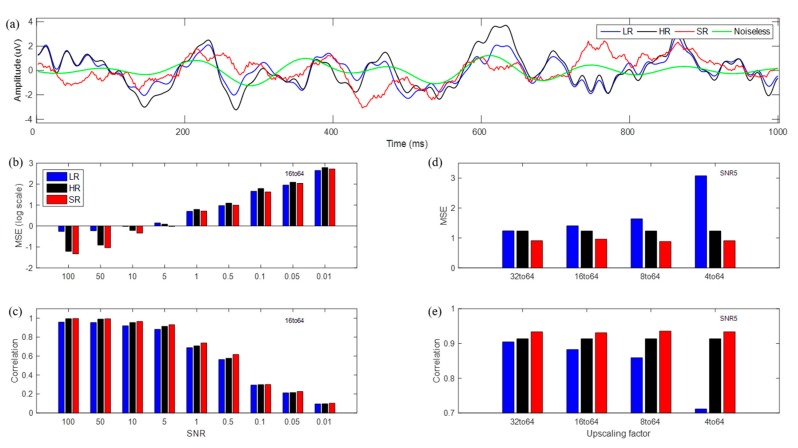
(**a**) Time series of LR (blue), HR (black), SR (red), and noiseless (green) signal for single trial with real noise with an SNR of 5 at CPz channel; (**b**) logarithm of MSE from noiseless signals; (**c**) correlation values according to an SNR with 16→64 up-scaling factor; (**d**) MSE, and (**e**) correlation values according to up-scaling factors with an SNR of 5.

We investigated SR methods for simulated data with real brain noise, as shown in Figure 7. HR time series have larger errors for brain noise than those of white Gaussian noise at the same SNR. Brain noise has a larger power in the low frequency than in the high-frequency band, while white Gaussian noise has uniform power over all frequency bands. Although brain noise yielded a lower correlation than did white Gaussian noise, SR demonstrated better or comparable performance in MSE and correlation compared to LR and HR. However, performance differences in real brain noise became far smaller than those in white Gaussian noise, and we also observed that SR data reduced noise, but the reduction was not notably large. At an SNR of 5 with the 16→64 up-scale, SR that yielded 30% fewer errors than LR was estimated roughly from actual MSE values (LR: 1.41, SR: 0.96) and SR with a correlation 5% higher than that of LR was estimated from actual correlation values (LR: 0.88, SR: 0.93).

**Figure 8:**
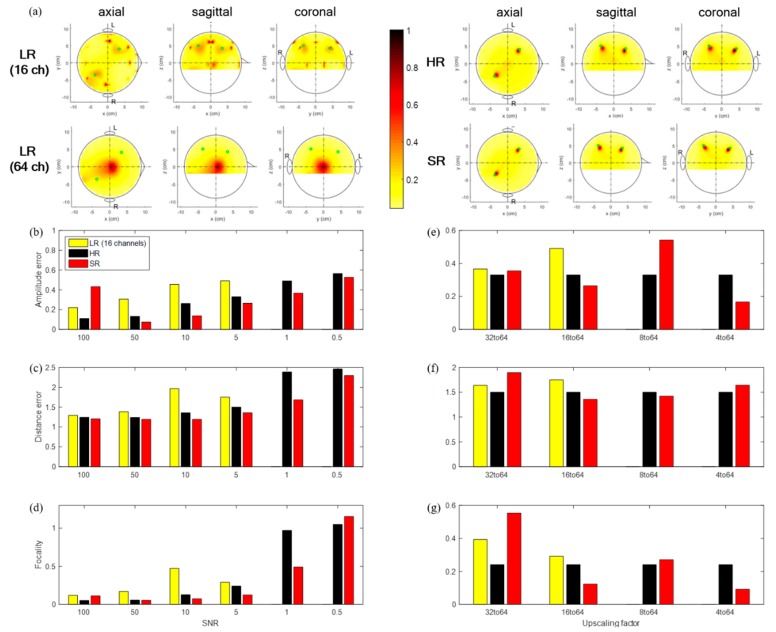
(**a**) Topographic of source localization using LR (16 channels; no interpolation), interpolated LR, HR, SR signal for single trial with real noise with an SNR of 5, exact source location (green dot); (**b**,**c**,**d**) amplitude error, distance error, and focality according to an SNR with 16→64 up-scaling factor; (**e**,**f**,**g**) amplitude error, distance error, and focality according to up-scaling factor with an SNR of 5.

Figure 8 shows the source localization results for simulated data with real brain noise. SR seemed to perform comparably to LR or HR or slightly more poorly. However, LR could not detect the sources using cases with and without interpolation for the 8→64 and 4→64 scale-ups, while SR data did. In addition, HR and SR data detected sources for high SNRs of 5 or above. However, they identified three sources (two brain sources and one noise source) for an SNR of 1. Overall, although the SR approach with simulated data with real brain noise demonstrated slightly poorer localization performance than with simulated data with white Gaussian noise, the SR approach may work reasonably well with real brain noise, and thus, these data may recover important original information better than LR data or as well as the original data (HR). SR had 45% and 22% fewer errors in amplitude and localization error, respectively, and SR was 11 times more focal to sources than without interpolation LR (LR without interpolation: 0.49, 1.75, 1.29, SR: 0.27, 1.36, 0.12) for the 16→64 up-scale at an SNR of 5.

### 3.3. SR Results for Experimental AEP Data

**Figure 9:**
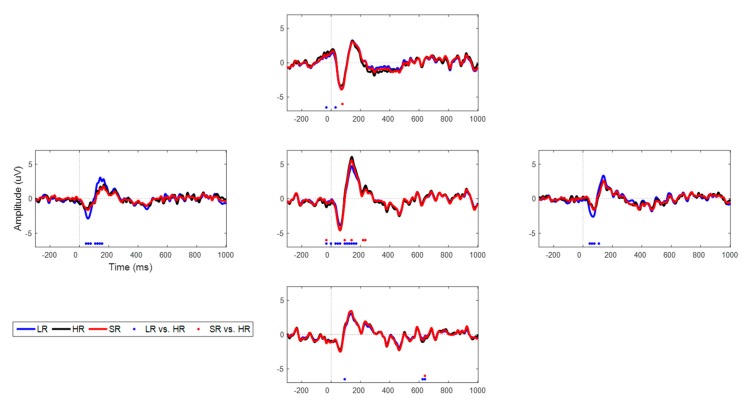
Time series of LR (blue), HR (black), and SR (red) for auditory evoked potential (AEP) data (one representative set of five sets) at the AFz, TP7, CPz, TP8, and POz channels with 16→64 up-scaling factor. Dotted marks are time points that differ statistically (*p* < 0.01) from HR.

In addition to the simulated data both with white Gaussian and real brain noise, we explored the SR approach with the experimental AEP data. Specifically, we focused on N1 and P2 components, which are the most important characteristics in the AEP task, as illustrated in Figure 9. The detailed information on each component’s latency amplitude is tabulated in Table 1. We observed that latency errors were minuscule (≤10 ms) in both components; however, we could not find any trend in latency over various scale-up factors. Overall, LR and SR data behaved similarly to HR data, except for some sensors that were placed on the margins; those were interpolated with only a few sensors. SR followed the original data better, particularly temporal sensors that evidently have a larger N1-peak-to-P2-peak amplitude with LR data than in HR’s amplitude. The statistically different time points (uncorrected, *p* < 0.01) are marked in red or blue dots on the time axis. We observed that LR differed statistically from HR at far more time points than did SR from HR. In particular, LR yielded statistically different points at the CPz, TP7, and TP8 channels, while SR did so only at the CPz channels. Overall, we found that SR data followed the HR data more closely in a statistical sense, although LR and SR exhibited similar behavior at the AFz and POz channels.

**Figure 10:**
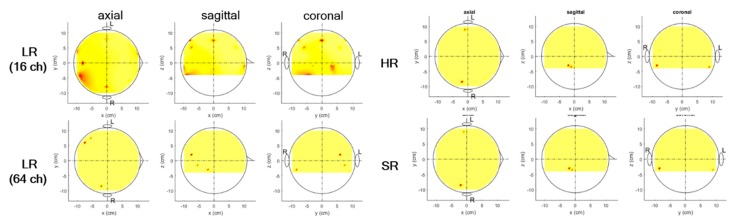
Source localization results for AEP data with 16→64 up-scaling factor (one representative set of five sets).

Source localization for N1 and P2 components was performed together, and voxels activated over the given power threshold (0.1) were estimated for each. We observed that two sources on both auditory cortices (one on each hemisphere) were identified with the original HR experimental data, which is believed to serve as ground truth (Figure 10). LR data without interpolation (16-channel LR) failed to detect any correct sources, while they identified three with interpolation (64-channel LR); however, two sources on the left hemisphere may have derived from a mismatch between LR and HR data in all scale-ups. The correct source was detected with weaker activations than ground truth (HR). The SR data identified the same two sources as those of the HR data, although the amplitudes of the sources detected differed slightly. However, SR data detected several spurious sources (which may explain noise information or even the small mismatch between SR and HR data) among some of the five sets for cross-validation that mentioned at the Section 2.2.

## 4. Discussion

### 4.1. Interpretation of SR Approaches at Sensor and Source Levels

In this work, we investigated the SR approach’s effect extensively using the deep learning technique for simulated and experimental data, and compared SR data with the conventional interpolated LR data and original HR data. The SR data far outperformed the others in most respects with simulated data with white Gaussian noise. Specifically, SR data reduced white Gaussian noise and improved the SNR. We also observed a similar noise reduction effect for simulated data, even that with real brain noise, although it was marginal.

At the sensor level, the LR data showed relatively small MSE for quite low SNRs, indicating that the simple interpolation approach remains sufficiently good. However, MSE was zero or low for LR channels that overlapped with HR channels, and the MSE overall may be lower than with sophisticated SR learning depending on the SNRs. Because of MSE’s problems, we also calculated correlations, and that of LR was lower than that of SR. However, it was clear that SR data improved at the sensor level at all SNRs. In addition, LR with interpolation did not yield any reasonable results at the source level, while it showed detection ability in some cases without interpolation. However, LR without interpolation yielded weak activated sources over quite a broad area, including ground truth, as shown in Figure 8a. It is understood that their information may have very limited ability to yield strong sources uniformly over various SNRs, and thus, depending on cases, they either detected sources occasionally or did not. In contrast, the sophisticated deep learning approach recovered source information far better than did the simple interpolation approach, even HR. We observed that the SR approach may cancel out HR data’s noise characteristics, and thus, CNN output is likely to have higher SNRs than input data. Thus, SR data may yield more apparent sensor and source features, including amplitude, latency, localized sources, and focality than LR data and even HR data. Based on this observation, we expect that the SR method may make it possible to recover important features of EEG data even from only a few sensors and potentially reduce some high frequency (>30 Hz) noise.

### 4.2. Validation of Simulated and Experimental Data

We observed that SR data outperformed LR with simulated data, and even the original HR data. However, with the experimental AEP data, SR data estimated ERP amplitudes comparably well or only slightly better than did LR data. In the simulated data, we investigated a large number of single trials because we knew the ground truth (noiseless signal) for various SNRs and scale-ups. However, with the experimental AEP data, we used data averaged over trials rather than single trials because single trial EEG data are difficult to address directly. We note that single trial data with a given SNR (δ) are approximately similar to average data with an estimated SNR (δ/√N) for N trials. According to this reasoning, we investigated N1 and P2 ERP components (average data over all trials) at the sensor level because these ERP components are typical characteristics of AEP.

**Figure 11:**
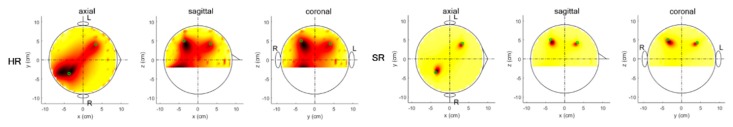
Topographic of source localization using HR, SR signal for single trial with white Gaussian noise with SNR of 0.5, and exact source location (green dot).

In addition, there is no ground truth in experimental data, so alternatively, HR (original EEG data) were considered ground truth, while noiseless EEG data were considered ground truth in simulated data. Noise definitely may contaminate HR data severely; in fact, HR could detect neither mismatched sources nor any sources at low SNRs because of their contamination, as shown in Figure 11. Thus, in reality, HR training of noise information is not recommended in the SR approach. However, in this study, we found that the SR approach may recover important characteristics of EEG data even from a limited number of sensors.

As expected, we observed that SR data provided reasonable source localization in two brain regions (auditory cortices) [45,47] in most cases. However, LR data with or without interpolation did not yield reasonable sources except for quite high SNR cases with the simulated data. AEP sources are known to be located farther away than are simulated sources, and the two different signals affected the central sensors (CPz) less than did the simulated data (Figure 1). Thus, experimental AEP data may yield slightly different results than simulated data (Figure 6a, Figure 8a).

Despite the differences between the simulated and experimental data, we compared them according to the given SNR. Experimental data could not separate signal and noise terms, and thus, the experimental data’s SNR was estimated as the power of the post-stimulus (0–1 s) divided by that of the pre-stimulus (−0.3–0 s) for the sensor level. The experimental data’s estimated SNR was approximately 1.2. Compared to simulated data with brain noise at an SNR of 1, experimental data yielded similar MSEs of approximately 1 (log scale) and a slightly larger correlation. In addition, in source localization, the estimated SNR was approximately 1.1, which was estimated as the power of the N1 and P2 components (0.2–2.5 s) divided by the power of the pre-stimulus (−0.3–0 s) because we computed an inverse operation using the N1 and P2 components for source localization. The experimental data’s amplitude error was lower than 0.3, which is smaller than the amplitude error of simulated data with brain noise for SR.

### 4.3. Source Localization Results for Experimental AEP Data

In simulated data, clear source information was reconstructed in the SR data and was more focal than in the HR data, while LR data could not identify any reasonable sources. The SR data detected sources well in the experimental AEP data, which were nearly identical to the sources the HR data detected (assumed ground truth). It is interesting to note that even the LR data demonstrated reasonable source detection in addition to spurious and weak sources in the left hemisphere. We also investigated the spurious sources’ origin. Based on source localization of temporal window data between −50 to 0 ms before onset, we found sources on the left hemisphere similar to those of LR data’s N1 and P2 components. Thus, we believe that the spurious sources may derive from noise information. We observed that some SR data for the 4, 8, and 16→64 scale-ups demonstrated one spurious source quite similar to one of the LR-estimated spurious sources. Thus, we determined that deep CNN learned noise information and SR data may contain such noise in the N1 and P2 components. In addition, we noticed that the SR data’s AEP time series showed certain other peaks than those in the LR and HR data. We expect that our proposed CNN may be able to be tuned further, and thus, yield better results, which will be investigated in a subsequent study.

### 4.4. Enhancing EEG Spatial Resolution Methods

Surface interpolation or cortical imaging methods have been applied to improve EEG’s spatial resolution [10,11,12,13,14,15,16,17,18,19,20,21,22,23,24,25,26,27]. However, artefacts from a single noisy sensor or eye movements influence surface interpolation, and it also is sensitive to spline parameters [17,21]. In addition, the cortical imaging method depends strongly on the volume conductor model [26]. Recently, compressed sensing that recovered original data with fewer features was reported; however, the approach may be applied only when the assumption of data redundancy is satisfied [28,29]. Thus, despite the necessity for high-density EEG systems and conventional methods’ disadvantages, virtually no new approaches have been developed in the past several years.

In this work, we investigated the possibility that SR using CNN can enhance EEG’s spatial resolution. SR has less model dependency attributable to its ability to learn HR data without a head model. In addition, resolved signals are far more robust to brain noise than are simple interpolated data, and previous work has shown that distortion in non-brain signals can be removed by applying a pre-whitening method [33]. With improved spatial resolution, EEG signals could be obtained from only several sensors if HR data were recorded once to construct the model. Further, SR may be very promising when used to construct a subject-independent model using numerous subjects’ data.

### 4.5. Activation Functions

**Figure 12:**
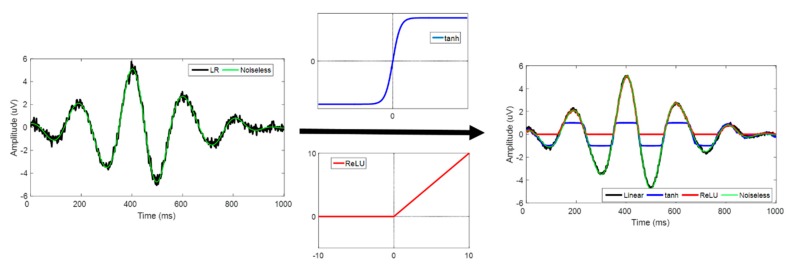
Time series of SR single trial data that were activated by linear (black), hyperbolic tangent (blue), and ReLU (red) function at an SNR of 100. Noiseless data are shown in green.

Conventional deep CNN applies non-linear functions as activation functions on convolutional layers, such as the rectifier (ReLU) and hyperbolic function (tanh). However, our proposed model was designed with a linear function (*y = x*). Our problem was finding the optimal fit line that minimizes the MSE, and the rectifier function was activated with zeros for negative values. An image consists of pixels that range from 0 to 255; however, EEG data range from negative infinite to positive infinite, and ReLU may not cover brain signals’ negative ranges. The hyperbolic function is slightly more flexible, but its optimal line is limited to the range from −1 to 1 (Figure 12). Although a normalizing technique could be an effective way to apply the function, we observed that it also transformed the data covariance’s properties, and transformed data may include localization error. Thus, the EEG SR problem should be distinguished from the classification problem, particularly when data are processed at the source level.

### 4.6. Study Limitations

In this study, we demonstrated SR techniques’ feasibility and efficacy with EEG data using our designed deep CNN. However, there were some limitations.

First, we designed a deep CNN structure for this SR purpose and considered many parameters in its structure. We note that our CNN was designed very carefully and our kernel configuration and other parameters were determined empirically. Seeking optimal CNN models is very compelling, although we confirmed that our proposed CNN model works reasonably well for our purpose. Among the parameters, we applied various stopping criteria for training (40 to 500 iterations) depending on the SNR and spatial resolution upscaling, because the error level achievable varies over the given data. Thus, it was quite difficult to set a universal stopping criterion, such as the error level, error difference between previous and present steps, iteration numbers, and so on. When the number of iterations was fixed to 50, as in previous work [33], we observed that CNN training seemed incomplete, and some of the SR data channels did not follow the trends of noiseless data even if they were superior to LR and HR. More iterations (over 100) demonstrated larger MSE than the fixed iteration (50) with a low SNR; however, over 100 iterations yielded a higher correlation with noiseless data than did 50 iterations. Therefore, more iterations may be necessary to achieve better results, in that SR data may follow the trends of clean data well, as well as high-frequency information, although they have larger MSEs than do fixed iterations. In addition, there may be better CNN structures for the SR purpose, which we still are seeking. Adding residual and dense blocks to the CNN structure considers hierarchical features [6], and thus, may enhance SR performance.

Second, when generating simulated data, we considered two sources that are quite far apart. This is a specific case and cannot be generalized. However, it is still sufficiently good that this dual-source problem may mimic the experimental AEP data; thus, through our extensive investigation with two-source simulated data, we may speculate the way the SR technique using CNN works for AEP experimental data. Simulated data commonly are generated simply by adding the given noise (acquired real brain noise or colored or white noise) to the EEG data computed in EEG simulation studies. It definitely is possible to generate colored noise from numerous spurious sources distributed randomly at the source level. However, in this work, we judged that real brain noise is more realistic than any colored noise because real brain noise is obtainable. In any case, we are investigating various other cases currently to identify more ways to apply the SR technique in EEG.

Third, we verified CNNs’ feasibility for EEG SR using AEP experimental data from one subject. Thus, this work may be limited, as no statistical analysis over subjects was conducted to determine its inter-subject variability. In practice, such variability is an important issue; thus, it is necessary to perform statistical tests on data from a large number of subjects. However, our goal in this study was to investigate CNNs’ feasibility through an extensive simulation study and validate it with experimental data. Investigation of inter-subject variability and development of a subject-independent SR model by CNN will be an interesting issue to pursue in future work.

Fourth, a CNN may capture local spatial features and apply the same weight over neighboring spatial and temporal regions, in that the input channel order may affect output matrices used to compute the convolution operation. Our channel ordering was set according to the Biosemi system’s channel configuration (frontal–central–temporal–parietal–occipital in the left hemisphere, occipital–central–frontal at the midline, and frontal–central–temporal–posterior–occipital in the right hemisphere). Neighboring EEG sensors have similar electrical potentials because they are blurred by the skull [26] and convolutional operator, in which channel clusters with similar values may be learned more effectively. Wen et al., proposed an EEG channel reordering algorithm that maximizes adjacent information and reported that their channel ordering yielded higher classification accuracy than did other channel ordering [48]. However, in our channel ordering, CP1, CPz, and CP2 channels in convolution operations were computed with different weights. We expect that SR results are likely to be enhanced when neighboring channels are considered carefully.

Lastly, when we selected channels, we chose them according to the 10–10 international EEG system (Figure 2). In our selection, all channels were considered to be distributed evenly on the head. There may be numerous other selections than ours. We expect that our results may not differ very significantly if channel selection is not biased seriously in a specific region. Recently, ear-EEG systems [49,50,51,52] and frontal EEG systems [53,54] have been developed to overcome the inconvenience of the whole head experiment as well as reduce cost. Their feasibility has been tested with ERP components during an auditory task or alpha-attenuations during sleep or while playing games; however, they could not acquire EEG data from the entire scalp; thus, their applicability may be quite limited, as they could not estimate source information and functional connectivity between two different brain regions. With this reasoning, applying the SR technique with a biased selection of channels (recovering the entire scalp EEG or scalp on the motor area from just a few channels around the ears) may be quite interesting, which is now under investigation.

Although these various issues should be considered further to enhance EEG spatial resolution by CNN, we proposed the EEG SR method using CNN firstly and demonstrated its feasibility through simulated and experimental data. Specifically, we investigated our proposed method according to the aspects of sensor and source analyses; thus, we believe that SR data may be useful when investigating brain dynamics in both sensor and source spaces. Because connectivity studies have attracted more attention in neuroscience recently, our proposed method may be quite applicable in this respect.

## 5. Conclusions

In this work, we investigated SR techniques’ effects using deep CNN on EEG data with white Gaussian noise and real brain noise. In addition, we verified the deep learning models using experimental AEP data. Our results showed that SR data demonstrated higher performance than simple interpolated data (LR) or performance comparable to that of HR datasets, as they maintained signal properties. Therefore, the model can be applied in an environment in which high spatial resolution EEG data cannot be easily collected.

## Figures and Tables

**Table 1:** Amplitude and latency information on N1 and P2 components for LR, HR, and SR (means and standard deviations over five sets).

**N1 Component**		**CPz**		**AFz**		**TP7**		**TP8**		**POz**	
		**Amplitude** **(uV)**	**Latency** **(ms)**	**Amplitude** **(uV)**	**Latency** **(ms)**	**Amplitude** **(uV)**	**Latency** **(ms)**	**Amplitude** **(uV)**	**Latency** **(ms)**	**Amplitude** **(uV)**	**Latency** **(ms)**
32to64	LR	4.4 ± 0.3	67.0 ± 2.6	4.3 ± 0.6	70.8 ± 2.3	2.9 ± 0.6	68.8 ± 3.0	2.9 ± 0.3	72.0 ± 4.0	2.6 ± 0.3	68.2 ± 3.9
	HR	5.0 ± 0.3	65.8 ± 3.5	4.2 ± 0.7	68.8 ± 4.6	1.6 ± 0.4	61.8 ± 21.7	1.9 ± 0.4	72.8 ± 3.0	2.4 ± 0.3	68.8 ± 3.3
	SR	5.0 ± 0.4	66.6 ± 3.0	4.5 ± 0.6	71.6 ± 1.7	1.8 ± 0.5	68.2 ± 7.6	1.9 ± 0.3	71.6 ± 3.6	2.8 ± 0.3	67.4 ± 3.0
16to64	LR	4.5 ± 0.4	68.0 ± 2.8	4.4 ± 0.6	70.4 ± 3.0	3.3 ± 0.4	68.2 ± 4.3	3.1 ± 0.4	69.6 ± 3.3	2.5 ± 0.2	67.8 ± 3.5
	HR	5.0 ± 0.3	65.8 ± 3.5	4.2 ± 0.7	68.8 ± 4.6	1.6 ± 0.4	61.8 ± 21.7	1.9 ± 0.4	72.8 ± 3.0	2.4 ± 0.3	68.8 ± 3.3
	SR	5.0 ± 0.3	67.0 ± 3.3	4.4 ± 0.5	71.6 ± 2.2	1.8 ± 0.4	69.8 ± 5.1	2.0 ± 0.3	73.2 ± 3.0	2.7 ± 0.4	68.0 ± 2.4
8to64	LR	5.2 ± 0.7	68.4 ± 2.2	3.5 ± 0.5	71.6 ± 3.6	3.2 ± 0.4	68.2 ± 4.3	3.0 ± 0.4	70.8 ± 3.0	1.5 ± 0.3	67.4 ± 4.9
	HR	5.0 ± 0.3	65.8 ± 3.5	4.2 ± 0.7	68.8 ± 4.6	1.6 ± 0.4	61.8 ± 21.7	1.9 ± 0.4	72.8 ± 3.0	2.4 ± 0.3	68.8 ± 3.3
	SR	5.5 ± 0.7	68.2 ± 4.3	4.6 ± 0.6	73.2 ± 3.0	2.0 ± 0.4	68.2 ± 6.6	2.1 ± 0.4	73.2 ± 4.1	3.1 ± 0.2	68.2 ± 3.3
4to64	LR	2.2 ± 0.3	68.6 ± 5.0	2.9 ± 0.4	73.6 ± 4.3	2.1 ± 0.2	68.2 ± 6.5	2.0 ± 0.3	68.4 ± 4.1	2.2 ± 0.3	68.6 ± 5.0
	HR	5.0 ± 0.3	65.8 ± 3.5	4.2 ± 0.7	68.8 ± 4.6	1.6 ± 0.4	61.8 ± 21.7	1.9 ± 0.4	72.8 ± 3.0	2.4 ± 0.3	68.8 ± 3.3
	SR	4.0 ± 0.4	70.8 ± 2.7	4.2 ± 0.5	72.0 ± 1.4	1.8 ± 0.4	60.2 ± 10.6	2.3 ± 0.2	73.6 ± 5.9	2.8 ± 0.3	69.6 ± 3.3
**P2 Component**		**CPz**		**AFz**		**TP7**		**TP8**		**POz**	
		**Amplitude** **(uV)**	**Latency** **(ms)**	**Amplitude** **(uV)**	**Latency** **(ms)**	**Amplitude** **(uV)**	**Latency** **(ms)**	**Amplitude** **(uV)**	**Latency** **(ms)**	**Amplitude** **(uV)**	**Latency** **(ms)**
32to64	LR	5.0 ± 0.5	139.4 ± 4.8	2.9 ± 0.6	150.4 ± 8.8	2.6 ± 0.3	147.6 ± 10.9	2.6 ± 0.4	146.4 ± 12.1	3.3 ± 0.5	137.4 ± 3.8
	HR	6.1 ± 0.6	139.4 ± 4.8	2.9 ± 0.6	150.8 ± 8.7	2.0 ± 0.3	150.6 ± 11.2	2.3 ± 0.3	149.0 ± 12.9	3.5 ± 0.6	133.8 ± 5.4
	SR	5.5 ± 0.5	138.6 ± 5.5	2.9 ± 0.7	150.6 ± 8.7	1.7 ± 0.2	151.8 ± 12.0	2.4 ± 0.3	147.2 ± 10.3	3.7 ± 0.6	133.0 ± 6.3
16to64	LR	4.8 ± 0.5	140.2 ± 5.2	3.0 ± 0.6	149.6 ± 8.1	3.2 ± 0.4	143.0 ± 3.5	3.3 ± 0.4	137.4 ± 7.9	3.2 ± 0.5	137.8 ± 4.4
	HR	6.1 ± 0.6	139.4 ± 4.8	2.9 ± 0.6	150.8 ± 8.7	2.0 ± 0.3	150.6 ± 11.2	2.3 ± 0.3	149.0 ± 12.9	3.5 ± 0.6	133.8 ± 5.4
	SR	5.6 ± 0.6	139.4 ± 4.8	2.9 ± 0.7	151.2 ± 9.4	1.6 ± 0.2	152.2 ± 11.3	2.3 ± 0.3	144.2 ± 10.8	3.6 ± 0.5	132.6 ± 5.5
8to64	LR	4.7 ± 0.5	141.4 ± 4.8	2.2 ± 0.5	153.8 ± 12.2	2.9 ± 0.4	143.2 ± 2.7	2.9 ± 0.4	137.8 ± 8.3	2.2 ± 0.7	131.0 ± 7.6
	HR	6.1 ± 0.6	139.4 ± 4.8	2.9 ± 0.6	150.8 ± 8.7	2.0 ± 0.3	150.6 ± 11.2	2.3 ± 0.3	149.0 ± 12.9	3.5 ± 0.6	133.8 ± 5.4
	SR	5.0 ± 0.6	137.8 ± 6.1	3.2 ± 0.7	156.0 ± 12.0	1.8 ± 0.4	153.6 ± 11.4	2.3 ± 0.4	144.0 ± 13.9	3.4 ± 0.7	130.6 ± 6.2
4to64	LR	2.0 ± 0.3	143.8 ± 5.1	2.1 ± 0.4	153.6 ± 11.4	2.0 ± 0.3	143.4 ± 4.2	2.0 ± 0.4	136.6 ± 7.5	2.0 ± 0.3	143.8 ± 5.1
	HR	6.1 ± 0.6	139.4 ± 4.8	2.9 ± 0.6	150.8 ± 8.7	2.0 ± 0.3	150.6 ± 11.2	2.3 ± 0.3	149.0 ± 12.9	3.5 ± 0.6	133.8 ± 5.4
	SR	3.6 ± 0.8	137.4 ± 4.8	2.8 ± 0.6	149.0 ± 6.2	1.8 ± 0.3	150.2 ± 9.9	1.8 ± 0.3	145.4 ± 6.3	3.0 ± 0.7	132.6 ± 5.2
